# Maternal bonding behavior, adult intimate relationship, and quality of life

**DOI:** 10.1007/s40211-017-0258-6

**Published:** 2018-01-11

**Authors:** Michaela Schmoeger, Matthias Deckert, Petra Wagner, Ulrike Sirsch, Ulrike Willinger

**Affiliations:** 1 Department of Neurology, Medical University of Vienna, Waehringer Guertel 18–20, 1090 Vienna, Austria; 2 Department of Social Work, School of Medical Engineering and Applied Social Sciences, University of Applied Sciences Upper Austria, Linz, Austria; 3 Department of Applied Psychology: Health, Development, Enhancement and Intervention, Faculty of Psychology, University of Vienna, Vienna, Austria

**Keywords:** Maternal bonding behavior, Adult intimate relationship, Quality of life, Continuity versus discontinuity hypothesis, Mütterliches Bonding, Enge Beziehung im Erwachsenenalter, Lebensqualität, Hypothese Kontinuität versus Diskontinuität

## Abstract

Continuity and discontinuity in the development of social relationships have been investigated by reviewing the course of social bonds and by analyzing the effects of a sound intimate relationship in adulthood in conjunction with recalled maternal bonding on the quality of life among students.

A questionnaire-based study of 207 students was conducted. Perceptions of maternal bonding were designated as being representative of one of the two contrasting bonding types “optimal maternal bonding” and “affectionless maternal control” assessed by the Parental Bonding Instrument (PBI) and combined with perceptions of a sound intimate adult relationship measured by the Family Assessment Measure III Dyadic Relationships Scale (FAM-III-D). Quality of life and general health data were determined by using the World Health Organization Quality of Life (WHOQOL-BREF) instrument.

Students who reported “optimal maternal bonding” had intimate relationships in adulthood that were of significantly higher quality than those who recalled “affectionless maternal control”. Students who recalled “optimal maternal bonding” and described their intimate relationship as sound showed significantly higher scores in all domains of quality of life and indicated having better general health than those who reported “affectionless maternal control” and a sound intimate relationship.

A sound intimate relationship in adulthood does not appear to compensate the impact of a recalled maternal bonding behavior in terms of affectionless control, on quality of life. Furthermore, results seem to support the hypothesis of continuity of the development of social relationships among psychologically well individuals based on the association between maternal bonding and later intimate relationships.

## Zusammenfassung

Kontinuität und Diskontinuität in der Entwicklung sozialer Beziehungen wurden durch die retrospektive Betrachtung des Verlaufs sozialer Bindungen und dessen Auswirkungen auf partnerschaftliche Beziehungen im Erwachsenenalter sowie durch die Analyse von Zusammenhängen zwischen dem erinnerten mütterlichen Bonding und der Lebensqualität untersucht.

Es wurde eine Fragebogenstudie mit 207 Studierenden durchgeführt. Die Wahrnehmung des mütterlichen Bondings wurde als repräsentativ für eine der beiden kontrastierenden Bonding-Typen „optimales Bonding“ und „lieblose Kontrolle“ bestimmt (gemessen anhand des „Parental Bonding Instrument“, PBI) und mit der Wahrnehmung einer sehr guten partnerschaftlichen Beziehung im Erwachsenenalter kombiniert (erfasst anhand der deutschen Version des „Family Assessment Measure III-Dyadic Relationships Scale“, FAM-III-D). Die Lebensqualität und allgemeine Gesundheitsdaten wurden mit dem Instrument zur Erfassung der Lebensqualität der Weltgesundheitsorganisation (WHOQOL-BREF) erfasst.

Die Ergebnisse zeigten, dass partnerschaftliche Beziehungen im Erwachsenenalter von Studierenden, die über ein „optimales mütterliches Bonding“ berichteten, von bedeutend höherer Qualität waren als jene von Studierenden, die über „lieblose mütterliche Kontrolle“ berichteten. Studierende, die sich an ein „optimales mütterliches Bonding“ erinnerten und ihre partnerschaftliche Beziehung als sehr gut bezeichneten, zeigten signifikant höhere Werte in allen Bereichen der Lebensqualität und eine bessere allgemeine Gesundheit als diejenigen, die über eine „lieblose mütterliche Kontrolle“ und eine sehr gute partnerschaftliche Beziehung berichteten.

Eine sehr gute partnerschaftliche Beziehung im Erwachsenenalter scheint den Einfluss des mütterlichen Bondings im Sinne der „lieblosen Kontrolle“ auf die Lebensqualität nicht kompensieren zu können. Des Weiteren scheinen die Ergebnisse die Hypothese von der Kontinuität der Entwicklung sozialer Beziehungen psychisch gesunder Individuen basierend auf Grundlage der Assoziation zwischen mütterlichem Bonding und späteren partnerschaftlichen Beziehungen zu untermauern.

## Introduction

Bonding represents the emotional attachment between the primary caregiver and the child, appears shortly after birth, and implies a special and focused relationship towards the caregiver’s own offspring [1–3]. Bonding is a biological and adaptive process that warrants the satisfaction of the child’s needs for nurture and protection by its caregivers, primarily by the mother. The sensitivity of the mother in reading the signals made by the child, and an appropriate and quick response to these signals helps the child to develop a sense of trust in the world and in later social relationships. Individual differences in the security of child and parent relationships are thought to be important due to their implications for later development. Different patterns of attachment reflect the child’s different expectations or internal working models of the social world [1–3].

According to Bowlby [1–3], the development of social relationships is continuous. Early socialization experiences strongly influence any later capacity to form affectionate bonds. These experiences are gradually incorporated into the individual’s psychological organization and significantly influence the course of other relationships [4]. Childhood experiences are the principal determinants of abnormalities in adult relationships and can consequently lead to increased psychopathology [5]. Hazan and Shaver [6] suggested romantic love as an attachment process. Adults with insecure intimate relationships presented more negative expectations and beliefs about love, histories of shorter intimate relationships and less favorable childhood experiences with their parents than adults with secure intimate relationships. In subsequent research, disadvantages of insecure compared to secure attached individuals with respect to several aspects of adult intimate relationships have been reported (e. g. [7–9]).

In contrast, Belsky and Nezworski [10] argue that later social relationships may not be influenced continuously but may change in adulthood. The association between early bonding and attachment experiences and later social relationships may be described, according to Main Kaplan, and Cassidy [11], as being discontinuous. They reported that parents, who recalled their childhood attachments as insecure work cognitively through negative issues, become sufficiently secure in their own parenting skills and form secure attachments with their children. According to Parker et al. [5] any impairment in relationships between parents and children, except perhaps gross parental deprivation, seem to be subject to modification by a range of experiences, in particular subsequent interpersonal relationships.

Gittleman, Klein, Smider, and Essex [12] suggested that both continuity and discontinuity principles can be applied to understand links between early experiences with parents, adult relationships and adult mental health. They investigated associations of parental bonding behavior, adult attachment and adult mental health by considering independent, mediating, or moderating effects. They found patterns of moderating effects of adult relationship styles on the association between parental bonding behavior and adult mental health.

In the present study two research questions regarding the continuity versus discontinuity hypotheses of the development of social relationships were considered. First, the course of social relationships was investigated by testing the differences between two contrasting types of recalled maternal bonding behavior, namely “optimal maternal bonding” and “affectionless maternal control” [13], with respect to the soundness of adult intimate relationship. Second, a possible mediating effect of a healthy intimate relationship regarding the association between maternal bonding behavior and quality of life in adulthood was investigated. The two types of recalled maternal bonding behavior, “optimal maternal bonding” and “affectionless maternal control”, were combined with a sound intimate relationship in adulthood. If an intimate relationship buffers the effect of a negative maternal bonding type on quality of life, no differences between the two groups “optimal maternal bonding/sound intimate relationship” and “affectionless maternal control/sound intimate relationship” with respect to quality of life should occur.

## Methods

### Subjects

A sample of 207 students enrolled at the University of Vienna was recruited. The sample consisted of 171 (83%) female and 36 (17%) male students (CHI^2^ = 88.043; df = 1; *p* ≤ 0.0001). The students had been attending the university for a mean period of six terms (SD = 4.0); the mean age of the students was 24.64 years (SD = 6.27). 169 (82%) of the students were studying psychology, 38 (18%) were studying other disciplines. Being in a current intimate relationship was an inclusion criterion for participants. All subjects gave written informed consent prior to the study.

### Measures

Information about self-rated maternal bonding behavior during the first 16 years was assessed with the German version [14] of the Parental Bonding Instrument (PBI) [13], which contains two scales. Care was evaluated by 12 items on a continuum, ranging from empathy, closeness, emotional warmth, and affection to neglect, indifference, and emotional coldness. Control was measured by 13 items, ranging from overprotection, intrusion, excessive contact, control, infantilization, and prevention of independent behavior to autonomy and allowance of independence. Both the validity and the reliability of the PBI have been shown to be acceptable [13]. The scales of the PBI have been shown to be stable over time [15] and independent of current mood state [16] and the recipient’s gender [13].

Information about the quality of the intimate relationship was measured by the two “affective” [17] dimensions “involvement” and “affective expression” by means of the German version [17] of the Family Assessment Measure III Dyadic Relationships Scale (FAM-III-D) [18]. “Involvement” represents the quantity and quality of interest the partners have for each other. Emotional needs should be complementary in order to guarantee a feeling of security, a sense of togetherness and mutual esteem for appreciation of one another, a higher level of self-esteem, and independence. “Affective expression” reflects a dialectic relation between the security of the partnership and the autonomy of the individuals.

Information about quality of life and general health was gathered with the German version [19] of the World Health Organization Quality of Life (WHOQOL-BREF) instrument [20], which is composed of 26 items assigned to four domains. The physical domain represents constraints on quality of life by pain and discomfort, sleep and rest, energy and fatigue, mobility, activities of daily living, dependence on medicinal substances and medical aids, and work capacity. The psychological domain covers questions regarding positive feelings, thinking, learning, memory and concentration, self-esteem, body image and appearance, negative feelings, and spirituality/religion/personal beliefs. The domain social relationships reflects personal relationships, social support, and sexual activity. The domain environment is concerned with freedom, physical safety and security, home environment, financial resources, health and social care considering accessibility and quality, opportunities for acquiring new information and skills, participation in and opportunities for recreation/leisure activity, physical environment (pollution/noise/traffic/climate), and transport.

### Statistical analyses

Two groups of students were formed with respect to the two maternal bonding types and the quality of adult intimate relationship: one group with a recalled “optimal maternal bonding type” (high level of care and low level of control), combined with a healthy intimate relationship in adulthood, and the other with a recalled “affectionless maternal control type” (low level of care and high level of control), also combined with a sound adult intimate relationship. High levels regarding care and control refer to values above the median, low levels to values below the median. A sound adult intimate relationship is represented by values below the median on the two dimensions “involvement” and “affective expression”. Due to the reciprocal coding of the FAM-III-D, lower scores on these two dimensions indicate a sound intimate relationship.

Group differences between females and males within the student sample were tested for significance by chi-square tests. Group differences between the two groups of students were analyzed for significance by t‑tests. The cut-off level for statistical significance was set at *p* < 0.05, 2‑tailed. Alpha adjustment for multiple comparisons was applied using Holm’s Sequential Bonferroni Procedure [21]. All statistical analyses were performed by IBM SPSS Statistics 24.

## Results

Within the sample two maternal bonding types were distributed in the following way: 74 students (37%) recalled high levels of maternal care and low levels of maternal control, conceptualized as the “optimal bonding type” and 75 (37%) recalled low levels of maternal care and high levels of maternal control, conceptualized as “affectionless control”. The other two maternal types, not used for further analyses in this study, namely “affectionate constraint” and “absent of weak bonding” were equally shared by the remaining 56 (26%) students. (For more detail see [22]). According to the central limit theorem, normal distribution can be assumed for group comparisons [23]. A post hoc power analysis (N1 = 74, N2 = 75, d = 0.05) showed a power of 86% for the final sample size of 149 [24].

There were no significant differences between the two groups “optimal maternal bonding/sound intimate relationship” (sound “involvement” and sound “affective expression”, respectively) and “maternal affectionless control/sound intimate relationship” (sound “involvement” and sound “affective expression”, respectively) with respect to gender (CHI^2^ = 1.09; df = 1; *p* 0.297, CHI^2^ = 2.46; df = 1; *p* = 0.117, respectively). Consequently, the two groups were combined in the statistical analyses.

With respect to the first research question, significant differences were found between the two bonding types “optimal maternal bonding” and “affectionless control” regarding the two adult intimate relationship variables “involvement” (t = 2.7; df = 134; *p* = 0.007) and “affective expression” (t = 3.3; df = 112.7; *p* = 0.001). Students who reported an “optimal maternal bonding” showed a higher quality of “involvement” and “affective expression” in adulthood. Means and standard deviations are shown in Table 1.

**Table 1:** Means and standard deviations among the two groups “optimal maternal bonding” and “affectionless maternal control” with respect to “involvement” and “affective expression”

	Group 1 Optimal maternal bonding	Group 2 Affectionless maternal control
Involvement^a^	2.0 (±1.8)	2.9 (±2.0)
Affective expression^a^	1.1 (±1.4)	2.2 (±2.4)

^a^Lower scores indicate higher quality on both dimensions

As to the second research question, significant differences were found between the two groups “optimal maternal bonding/sound adult intimate relationship” and “affectionless maternal control/sound adult intimate relationship” with respect to all four domains as well as the “general health” section of the WHOQOL. Students who recalled an “optimal maternal bonding” and described their adult intimate relationship as healthy (sound “involvement” and sound “affective expression”, respectively) showed significantly higher scores in “general health” (t = 4.0; df = 49.3; *p* ≤ 0.0001, t = 3.7; df = 52.1; *p* = 0.001, respectively), in the “physical domain” (t = 2.5; df = 49.4; *p* = 0.016, t = 2.2; df = 76; *p* = 0.03, respectively), in the “psychological domain” (t = 3.7; df = 50.5; *p* ≤ 0.0001, t = 3.1; df = 50.2; *p* = 0.004, respectively), in the domain “social relationships” (t = 2.5; df = 79; *p* = 0.015, t = 3.5; df = 76; *p* = 0.001, respectively), and in the domain “environment” (t = 3.0; df = 78; *p* = 0.004, t = 2.9; df = 75; *p* = 0.006, respectively) in comparison to students who recalled “maternal affectionless control” and had a healthy intimate relationship. Results are significant at the *p* < 0.05 level after Holm-Bonferroni correction [21]. Means and standard deviations are shown in Tables 2 and 3.

**Table 2:** Means and standard deviations among the two groups “optimal maternal bonding/sound adult intimate relationship: intimate” and “affectionless maternal control/sound adult intimate relationship: intimate” with respect to quality of life

	Group I Optimal maternal bonding/sound adult intimate relationship: involvement	Group II Affectionless maternal control/sound adult intimate relationship: involvement
General health	87.2 (±13.3)	70.8 (±21.1)
Physical domain	89.3 (±8.5)	82.6 (±13.5)
Psychological domain	81.6 (±8.5)	71.8 (±13.1)
Domain social relationships	85.9 (±13.0)	77.5 (±17.4)
Domain environment	79.0 (±11.4)	70.2 (±15.0)

**Table 3:** Means and standard deviations among the two groups “optimal maternal bonding/sound adult intimate relationship: affective expression” and “affectionless maternal control/sound adult intimate relationship: affective expression” with respect to quality of life

	Group I Optimal maternal bonding/sound adult intimate relationship: affective expression	Group II Affectionless maternal control/sound adult intimate relationship: affective expression
General health	88.3 (±12.6)	74.2 (±19.0)
Physical domain	89.3 (±8.6)	84.3 (±11.3)
Psychological domain	78.9 (±10.5)	70.8 (±14.2)
Domain social relationships	86.2 (±12.7)	75.4 (±17.0)
Domain environment	79.0 (±11.4)	70.2 (±15.0)

## Discussion

Students who recalled “optimal maternal bonding”, defined by a high level of maternal care and low level of maternal control [13] indicated having intimate relationships in adulthood that were of significantly higher quality than students who recalled “affectionless maternal control”, defined by a low level of maternal care and high level of maternal control [13]. High levels of maternal empathy, closeness, emotional warmth, and low levels of overprotection, intrusion, excessive contact, control, and infantilization were related to an intimate relationship of high quality, defined by a high level of complementary emotional needs for both partners in order to guarantee a feeling of security, a sense of togetherness and mutual esteem for appreciation of one another, a higher level of self-esteem, independence, a high level of security within the partnership and high autonomy exhibited by both partners.

This result is in accordance with previously reported research findings showing that the perception of poor childhood support was related to lower sociability scores and a recalled lack of satisfactory peer relationships among students [25], that care scales (PBI) were associated with measures of social support made at the same time in a student group and that maternal care was also correlated with social satisfaction made one and a half years later [26], and that low levels of childhood care from both mothers and fathers were related to low levels of perceived social support among medical students [27]. Associations between parent–child bonding and later social bonding were rather found among those individuals who are psychologically well. However, these studies did not specifically investigate intimate adult relationships but rather social relationships in general. Some studies reported a lack of evidence to support the developmental continuity of social relationships (early and later ones) [28–31], especially for patients with a psychiatric disorder.

Moreover this result seems to support the hypothesis of continuity of the development of social relationships among psychologically well individuals on the basis of the association between maternal bonding and later intimate relationships. According to Bowlby [1–3] early bonding and attachment experiences are mentally represented as “internal working models” which influence the course of later social relationships. Children internalize experiences with caretakers in such a way that early attachment relationships come to form a prototype for later relationships. Different patterns of attachment reflect a child’s different expectations or internal working models of the social world. Internal working models include expectations about the caregiver’s availability, the infant’s own worthiness and ability to obtain care, and social relationships in general. Experiences of reliable and responsive care will lead to a model of the caregiver as available, of the self as worthy of care and effective in obtaining it, and of social relationships as pleasurable and rewarding [32].

Security of attachment is thought to be important due to its implications for later development [1–3]. Securely attached infants seem to be more socially competent, they tend to be more cooperative and obedient, they have better relations with their peers [33, 34] and they are less likely to develop emotional or behavior problems [35]. Children with secure attachment spend less time alone, less time with counselors, and more time with other children than do youngsters who had been attached insecurely as infants and toddler. Furthermore, they spend more time in groups of same-gender peers [36] and show more positive and less negative child–friend interactions [37]. They are less often ridiculed or excluded from group activities in middle childhood [38]. They tend to choose each other as friends, their friendships are likely to be reciprocal rather than one-sided, and a close friendship among secure children does not prevent them from functioning in the larger peer group. They often spend time together in groups, and their play together also includes other children [39]. Parental support, warmth, and authoritativeness are also connected with positive outcomes in adolescents [40].

Studies considering associations between early socialization experiences, quality of intimate relationships and mental health in adulthood concluded that the quality of current attachments might buffer the negative impact of low parental care on mental health [41]. Gittleman et al. [12] reported that adult attachment styles had few mediating effects on the association of parental bonding behavior and adult mental health. Interactions between the fearful style, parental care and control seem to accentuate the effect of high parental care or low parental control on mental health in both men and women. The secure adult relationship style seems to moderate somewhat the negative impact of high parental control in women.

The current study also analyzed the associations between early socialization experiences, defined as the combination of maternal care and control; adult intimate relationship, as reflected by the quantity and quality of interest exhibited by the partners for one another, security within the partnership as well as partner autonomy; and quality of life, whereby adult relationship could possibly moderate the effect of maternal bonding on quality of life. If a healthy adult intimate relationship compensates the effect of a negative maternal bonding type on quality of life, there should not be any differences between the two groups “optimal maternal bonding/sound intimate relationship” and “affectionless maternal control/sound intimate relationship” with respect to quality of life. However, in the present study, a healthy intimate relationship in adulthood did not appear to buffer the impact of recalled maternal bonding behavior characterized by a low level of care and high level of control, which Parker et al. [13] conceptualized as “affectionless control”, on the quality of life. Students who recalled an “optimal maternal bonding” and described their intimate relationship as being sound showed a significantly lower reduction of their quality of life on dimensions of pain and discomfort, sleep and rest, energy and fatigue, and higher values on parameters measuring positive feelings, thinking, learning, memory and concentration, self-esteem, bodily image and appearance, personal relationships, social support, sexual activity, freedom, physical safety and security than students with recalled maternal affectionless control and a healthy intimate relationship.

Gittleman et al. [12] found mediating and moderating effects in their study. The effect of maternal care on depression among women was mediated by the security of their closest relationships. Men and women with secure adult relationship styles showed low levels of distress regardless of levels of parental bonding behavior. Parental control among men or low parental care in combination with a fearful style were related to increased psychological distress. However, they also reported that while the fearful style was associated with higher levels of distress independent of recalled parental control, high levels of control, especially maternal control, were related to increased depression and anxiety among women with secure styles, although these levels never rose to the scores exhibited by those with fearful styles. Gittleman et al. [12] concluded that both continuity and discontinuity principles can be applied to the understanding of associations between early experiences with parents, adult relationships and mental health, and they suggested that it seemed to be more fruitful to consider the circumstances in which each applies rather than considering continuity and discontinuity as opposing hypotheses.

The results of the current study suggest support for the continuity hypothesis and show no buffer effect of a high quality intimate relationship in adulthood on the association of early maternal care and control and quality of life among psychologically well individuals. In short, the special circumstances in the present study were the effects social bonds had on both quality of life, instead of mental health as found in other studies, and on intimate adult relationships among psychological well individuals, instead of social support in general as determined in other studies.

Bowlby [42, p. 9] pointed out: “… I believe there is already sufficient evidence, […], that points to the very substantial influence on personality development and mental health of the way an individual’s parents (or in some cases parent substitutes) treat him or her”. In this context it seems that early childhood experiences of high-quality bonding do not only have an impact on personality development and mental health but also on the basic quality of life.

Nevertheless, some potential limitations of the current study concerning the sample must be taken into consideration. Participants were students, mostly psychology students, with a mean age of approximately 25 years. The selection of included subjects might have hand an influence on the outcome of the study. Psychology students might for example have higher interest in social interactions than the general population or show higher values of selflessness due to their biography. Both factors could have an impact on intimate relationships in adulthood. Furthermore, the age of the included subjects might indirectly influence the results because it can be assumed that many participants may not have experiences with very long-standing sound intimate relationships. The limited duration of sound adult intimate relationships might have influenced the results.

## References

[1] Bowlby J (1971). Separation.

[2] Bowlby J (1975). Separation, anxiety and anger.

[3] Bowlby J (1980). Loss.

[4] Sroufe L, Waters E (1977). Attachment as an organizational construct. Child Dev.

[5] Parker GB, Barrett EA, Hickie IB (1992). From nurture to network: examining links between perceptions of parenting received in childhood and social bonds in adulthood. Am J Psychiatry.

[6] Hazan C, Shaver P (1987). Romantic love conceptualized as an attachment process. J Pers Soc Psychol.

[7] Simpson JA (1990). Influence of attachment styles on romantic relationships. J Pers Soc Psychol.

[8] Collins NL, Feeney BC (2004). Working models of attachment shape perceptions of social support: evidence from experimental and observational studies. J Pers Soc Psychol.

[9] McCarthy G (1999). Attachment style and adult love relationships and friendships: a study of a group of women at risk of experiencing relationship difficulties. Br J Med Psychol.

[10] Belsky J, Nezworski T (1988). Clinical implications of attachment. Clinical implications of attachment.

[11] Main M, Kaplan N, Cassidy J (1985). Security in infancy, childhood, and adulthood: a move to the level of representation. Monogr Soc Res Child Dev.

[12] Gittleman MG, Klein MH, Smider NA, Essex MJ (1998). Recollections of parental behaviour, adult attachment and mental health: mediating and moderating effects. Psychol Med.

[13] Parker G, Tupling H, Brown L (1979). A parental bonding instrument. Br J Med Psychol.

[14] Willinger U, Heiden AM, Meszaros K, Formann AK, Aschauer HN (2002). Maternal bonding behaviour in schizophrenia and schizoaffective disorder, considering premorbid personality traits. Aust N Z J Psychiatry.

[15] Mackinnon AJ, Henderson AS, Scott R, Duncan-Jones P (1989). The Parental Bonding Instrument (PBI): an epidemiological study in a general population sample. Psychol Med.

[16] Wilhelm K, Parker G (1990). Reliability of the parental bonding instrument and intimate bond measure scales. Aust N Z J Psychiatry.

[17] Cierpka M, Die Familienbögen FG (1994). Ein Inventar zur Einschätzung von Familienfunktionen (B-Zweierbeziehungsfragebogen).

[18] Skinner HA, Steinhauer PD, Santa-Barbara J (1995). Familiy assessment measure – III manual.

[19] Angermeyer MC, Kilian R, Matschinger H (2000). WHOQOL-100 und WHOQOL-BREF. Handbuch fuer die deutschsprachige Version der WHO Instrumente zur Erfassung von Lebensqualitaet.

[20] WHOQOL Group (1998). Development of the World Health Organization WHOQOL-BREF quality of life assessment. The WHOQOL Group. Psychol Med.

[21] Abdi H, Salkind N (2010). Holm’s sequential Bonferroni procedure. Encyclopedia of research design.

[22] Schmoeger M, Deckert M, Sirsch U, Wagner P, Willinger U. Recalled maternal bonding and quality of life in young adults. Manuscript submitted for publication. 2017.

[23] Field A (2009). Discovering statistics using SPSS.

[24] Faul F, Erdfelder E, Lang AG, Buchner A (2007). G*Power 3: a flexible statistical power analysis program for the social, behavioral, and biomedical sciences. Behav Res Methods.

[25] Hojat M, Borenstein BD, Shapurian R (1990). Perception of childhood dissatisfaction with parents and selected personality traits in adulthood. J Gen Psychol.

[26] Sarason IG, Sarason BR, Shearin EN (1986). Social support as an individual difference variable: its stability, origins, and relational aspects. J Pers Soc Psychol.

[27] Flaherty JA, Richman JA (1986). Effects of childhood relationships on the adult’s capacity to form social supports. Am J Psychiatry.

[28] Hickie I, Parker G, Wilhelm K, Tennant C (1991). Perceived interpersonal risk factors of non-endogenous depression. Psychol Med.

[29] Hickie I, Wilhelm K, Parker G, Boyce P, Hadzi-Pavlovic D, Brodaty H (1990). Perceived dysfunctional intimate relationships: a specific association with the non-melancholic depressive subtype. J Affect Disord.

[30] Truant G, Herscovitch J, Lohrenz J (1987). The relationship of childhood experience to the quality of marriage. Can J Psychiatry.

[31] Truant GS (1994). Personality diagnosis and childhood care associated with adult marital quality. Can J Psychiatry.

[32] Bowlby J (1973). Separation.

[33] Fagot BI (1997). Attachment, parenting, and peer interactions of toddler children. Dev Psychol.

[34] Kerns KA, Bukowski WM, Newcomb AF, Hartup WW (1996). Individual differences in friendship quality: links to child-mother attachment. The company they keep: friendship in childhood and adolescence. Cambridge studies in social and emotional development.

[35] Erickson MF, Sroufe L, Egeland B (1985). The relationship between quality of attachment and behavior problems in preschool in a high-risk sample. Monogr Soc Res Child Dev.

[36] Elicker J, Englund M, Sroufe LA (1992). Predicting peer competence and peer relationships in childhood from early parent-child relationships. Family-peer relationships: modes of linkage.

[37] McElwain NL, Volling BL (2004). Attachment security and parental sensitivity during infancy: associations with friendship quality and false-belief understanding at age 4. J Soc Pers Relat.

[38] Grossmann KE, Grossmann K (1991). Attachment quality as an organizer of emotional and behavioral responses in a longitudinal perspective. Attachment across the life cycle.

[39] Shulman S, Elicker J, Sroufe L (1994). Stages of friendship growth in preadolescence as related to attachment history. J Soc Pers Relat.

[40] Sampson RJ, Laub JH (1994). Urban poverty and the family context of delinquency: a new look at structure and process in a classic study. Child Dev.

[41] Andersson L, Stevens N (1993). Associations between early experiences with parents and well-being in old age. J Gerontol.

[42] Bowlby J (1988). Developmental psychiatry comes of age. Am J Psychiatry.

